# Glances at the Hospitals

**Published:** 1903-04-25

**Authors:** 


### GLANCES AT THE HOSPITALS.

### VICTORIA INFIRMARY, GLASGOW.

The ordinary income amounted to £8,375, and the ordinary expenditure to £11,835. The extraordinary income amounted to £5,982, and the expenditure to £8,515. These taken together show that the total income was £14,357, the total expenditure £20,350, and that an adverse balance of £5,993 was left at the end of the year, which had to be met by drawing on the capital account; but as the revenue from investments only comes to £1,440 a year, it is evident that the capital account will not admit of such an inroad upon it for many years in succession. The Governors say that an ordinary income of fully £15,000 a year is necessary to maintain the infirmary and its allied institutions in full working order, and unquestionably this is so. Therefore either more money must be forthcoming or patients refused admission or expenses curtailed in some way. As the infirmary seems to be kept at a high standard, and relieves much distress in its district, it is to be hoped that the income will be forthcoming. The average daily number of patients was 162; the cost per patient was £6 1s. 2d.; the cost per bed occupied was £73 1s. 2d., and the average residence in the hospital was nearly 32 days, which strikes us as being a high average. At the end of the previous year 98 patients were in residence, and during the year of the report under notice 1,207 were admitted, making a total of 1,305 under treatment. Of these 112 remained in the hospital, 1,193 were treated to a conclusion, and 192 died, showing a mortality of 10·7 per cent.; but if 66 dying within 48 hours of admission be omitted, the mortality would fall to 7·3 per cent. The medical and surgical tables have columns showing the total numbers treated for the various diseases, the numbers recovered or relieved, and the number of deaths; hence we can pick out any particular disease at a glance and see the results. For instance, of acute rheumatism there were 38 cases and no death; of 54 cases of consumption, 42 were cured or relieved and 12 died; of 60 cases of pneumonia, 42 recovered and 18 died; of 38 cases of appendicitis, 32 recovered and 6 died. We regret to notice that a table showing the anæsthetic used during surgical operations is not given.

### NORFOLK AND NORWICH EYE HOSPITAL.

This report refers to the 81st year of the existence of the institution, The finances are in a satisfactory condition. A donation of £500 from the Earl of Leicester was received, as well as smaller sums from the executors of Mr. Stannard and

Mrs. Knights. At the beginning of the year there were 8 patients in residence, and 146 were admitted, making a total of 154 under treatment. Of these 40 were cured, 34 relieved, 4 not improved, 68 were made out-patients, and 8 remained under treatment. The above is an abstract of the only medical table printed, and no details of the various diseases are given, so that for purposes of comparison the report is useless.

